# Supplementary material for: Learning curve and surgical outcome of robotic assisted colorectal surgery with ERAS program
Source: Sci Rep. 2022 Nov 29;12:20566. doi: 10.1038/s41598-022-24665-w (PMC9709162; doi:10.1038/s41598-022-24665-w)
Supplement: Supplementary file 1 — Supplementary Information 1. [file 41598_2022_24665_MOESM1_ESM.zip › LSC Raw data-Ñ[▒K/090/H_ñw╝╨░O▒Kñσ.pdf]

正式摘要(電子簽章：000000199109276)

W55 / W55 ☐英文標題(注意:無英文姓名-請補病人基本檔!) ☐【讀取】憑證

臺中榮民總醫院

出院病歷摘要

轉入號 : 轉出號 :

[redacted]

(9)入院日期	109/09/09 (2020) 直腸外科 W55 - 141
(10)轉科日期	
(11)出院日期	109/09/17 (2020) 住院天數計 8 日

診斷

- 入院 :Colon cancer, cT4a N1a M0, Stage IIIB
  , s/p HDFL week 1 (2020/04/13), FOLFIRI (2020/4/21, 5/5, 5/18, 6/1)
  , s/p RT since 2020/4/29-2020/06/02, 25 times with dose 4500 cGy
- 出院 :Colon cancer, cT4a N1a M0, Stage IIIB
  , s/p HDFL week 1 (2020/04/13), FOLFIRI (2020/4/21, 5/5, 5/18, 6/1)
  , s/p RT since 2020/4/29-2020/06/02, 25 times with dose 4500 cGy , s/p Laparoscopic low anterior resection, end-to-end anastomosis + loop ileostomy + bladder repair on 2020/9/10

主訴

- for operation

病史

- This is a 56 y/o male with history of colon cancer, cT4a N1a M0, Stage IIIB.At first, he suffered from fever for 3 days and progressive anal pain with watery stool, then he went to our ER for help. Then, he was admitted to HEME ward for neoadjuvant CCRT. After complete the course of CCRT, follow up pelvis MRI showed partial response of primary tumor . This time, he was admitted for surgical treatment.

體檢發現

- Colon cancer, cT4a N1a M0, Stage IIIB, s/p HDFL week 1 (2020/04/13), FOLFIRI (2020/4/21, 5/5, 5/18, 6/1), s/p RT since 2020/4/29-2020/06/02, 25 times with dose 4500 cGy
  DATE CEA

  1090311 161.1
  1090410 196.1
  1090609 43.06
  1090630 No side effect
  1090805 No side effect
  - General appearance :
    - easy looking , conscious clear , without cardiopulmonary distress
    - Vital signs自動帶入date 體溫(BT) 脈搏(P) 呼吸(R) 血壓(BP) 身高(BH) 體重(BW) BMI
      20200909 36.6℃ 61次/分 18次/分 142/97mmHg 169.3cm 65.8kg 23.0
    - Vital signs: B.P. 142/97 mmHg , P.R. 61 /min , R.R. 18 /min , B.T. 36.6 ℃
  - HEENT :
    - Eyes: no anemic conjunctiva, no icteric sclera, normal EOM
    - ENT: no otorrhea, no rhinorrhea, no erythematous mucosa, no injected tonsil, no deformity
  - Neck: supple, freely movable, trachea midline,no deformity, no engorged jugular vein , no palpable thyroid or lymph node enlargement
  - Chest:
    - Inspection: Grossly normal, symmetric and fully expansion, no accessory muscle used, no operation scar, no spider angiomata, no gynecomastia
    - Palpation: no palpable mass or nodule, no subcutaneous emphysema, no tenderness

-
  - Ausculation: clear breathing sound
- Heart:
  - Palpation: no thrill, no heave
  - Percussion: normal heart size
  - Ausculation: regular heart beat, normal S1, S2, no gallop, no murmur
- Extremities: No deformity, full ranged of motion without limitation, no clubbing finger or nail cyanosis, no pitting edema, no ulcer or pigmentation, no obvious skin lesion, no varicose vein, no bone, joint or muscle tenderness

CRS CONDITION

- Karnofsky performance status: (100) Normal, no complaints, no evidence of disease
- PE of Abdomen:
- CEA: 1.41 ng/ml
- Imaging Studies:
- Colonoscope report: .
- Figures:
  1. colon & rectum

手術日期及方法

- 手術日期及方法20200910 1. Laparoscopic low anterior resection, end-to-end anastomosis, EndoGIA 60/3.8mmX5, CEEA- 29mm. Bilateral doughnut: intact, air tight test: done, no air leak.
  2. High ligation (+) below the left colic artery.
  3. Cut margin: proximal 15 cm, distal 3 cm.
  5. Take down of splenic flexure: (-).
  6. A 12mm penrose drain placed in the pelvis.
  7. Loop ileostomy was done over RLQ
  8. Urinary bladder perforation was noted and primary repair was done

住院治療經過

- After admission, operation was done on 9/10. Empiric antibiotic with ertapenem was used for peritonitis. We let him try water then diet. He can tolerance Bland II diet now. Under stable condition, we arranged discharge for him.

合併症

一般檢查

- 累積報告 :

DATE	TIME	ALB	TP	BIL.T	BIL.D	ALKP	AST	ALT	LDH	CHOL	TG	HDL-C	LDL	UA	NH3	AMY
1090909	2027									129	54		73	3.9		
1090909	2028	3.6	6.6	0.4	0.1	91	17	12	168							
1090910	1921	3.1		0.8	0.2	69	24	24								
1090911	0432	3.4		0.8				22								
1090914	0929						12	8								
1090917	0947	2.5		0.50	0.18		11	4								

DATE	TIME	WBC	RBC	HGB	HCT	MCV	PLT	BLST	NEUT	BAND	SEG	LYM	MON	EOS	BAS
1090909	2025	2520	3.58	10.7	33.9	94.7	164		59.1			27.8	7.9	4.8	0.4
1090910	1921	2080	3.69	11.1	34.2	92.7	147			1	81	15	3		
1090911	0431	2050	4.38	13.1	40.7	92.9	153		86.3			9.8	3.9	0.0	0.0
1090914	0943	7630	3.47	10.4			139				87.0	8.0	5.0	0.0	0.0
1090917	0856	9710	3.03	9.4	28.9	95.4	199		84.7			6.9	6.3	1.5	0.6

DATE	TIME	NA	K	CL	CA	BUN	CREAT	Mg	P	CRP	ESR	PCT	LIPASE	LACTATE
1090909	2027									0.032				
1090909	2028	143	4.0	108	9.4	12	0.83							
1090910	1921	140	2.8	105	8.2	9	0.72							
1090911	0432	139	4.2			10	0.89			4.328				46.1
1090911	1535													24.1
1090914	0720													4.8
1090914	0929	137	3.4		8.3	12	0.73							
1090917	0947	137	3.8		8.0	6	0.70							

DATE	TIME	GLUCOSE	PAB	TRF	ZN	CU	KETONE	CK	CKMB	PT-P	PT-C	APTT-P	APTT-C	TRO-T
1090909	2025	87								10.7	10.3	27.1		
1090910	1921	179												

DATE	TIME	PH	BEB	SCVO2	PO2	HCO3	HGB	PCO2	TCO2

1090911	1538	7.334	-1.4	34.9	23.1	25.0		48.0	26.5
1090914	0718	7.415	0.3	87.1	52.1	24.7		39.5	26.0

特殊檢查

病理報告

其它

出院時情況

- 3.改門診治療

出院指示

藥名	劑量	劑量單位	用法	頻次	數量	數量單位	現況	天數	單位
Pantoloc tab 40mg	1	TAB	PO	QDAC	13	TAB	TH	13	天
Ultracet tab	1	TAB	PO	Q6H	52	TAB	TH	13	天
C.B. oint 5gm		TUB	TOP	TID	0	TUB	TH	1	瓶
12μg Durogesic patch	1	TAP	TOP	Q3D	13	TAP	TH	13	天

出院計劃

主治醫師 2062F 蔣鋒帆 / 住院醫師 2330E 楊捷儒

正式摘要(電子簽章：**000000199109276)**

W55 / W55

doctorRank:B
